# Associations between sleep bruxism and (peri-) implant complications: a prospective cohort study

**DOI:** 10.1038/bdjopen.2017.3

**Published:** 2017-04-14

**Authors:** Magdalini Thymi, Corine M Visscher, Eiko Yoshida-Kohno, Wim Crielaard, Daniel Wismeijer, Frank Lobbezoo

**Affiliations:** 1 Section of Oral Kinesiology, Department of Oral Health Sciences, Academic Centre for Dentistry Amsterdam, Amsterdam, The Netherlands; 2 Removable Partial Prosthodontics, Oral Health Sciences, Graduate School of Medical and Dental Sciences, Tokyo Medical and Dental University, Tokyo, Japan; 3 Section of Preventive Dentistry, Department of Oral Health Sciences, Academic Centre for Dentistry Amsterdam, Amsterdam, The Netherlands; 4 Section of Oral Implantology and Prosthetic dentistry, Department of Oral Health Sciences, Academic Centre for Dentistry Amsterdam, Amsterdam, The Netherlands

## Abstract

**Objectives/Aims:**

To describe the protocol of a prospective cohort study designed to answer the question: ‘Is sleep bruxism a risk factor for (peri-)implant complications?’.

**Materials and Methods:**

Our study is a single-centre, double-blind, prospective cohort study with a follow-up time of 2 years. Ninety-eight participants fulfilling inclusion criteria (planned treatment with implant-supported fixed suprastructure(s) and age 18 years or older) will be included. Sleep bruxism will be monitored at several time points as masticatory muscle activity during sleep by means of a portable single-channel electromyographic device. Our main outcomes are biological complications (i.e., related to peri-implant bleeding, probing depth, marginal bone height, quality of submucosal biofilm and loss of osseointegration) and technical complications (i.e., suprastructure, abutment, implant body or other).

**Results:**

The study is currently ongoing, and data are being gathered.

**Discussion:**

The results of this prospective cohort study will provide important information for clinicians treating bruxing patients with dental implants. Furthermore, it will contribute to the body of evidence related to the behaviour of dental implants and their complications under conditions of high mechanical loadings that result from sleep bruxism activity.

**Conclusion:**

The protocol of a prospective cohort study designed to investigate possible associations between sleep bruxism and (peri-) implant complications was presented.

## Introduction

### Implant treatment complications

Treatment with dental implants is one of the important options for patients with a partially or completely edentulous jaw. Dental implants are installed in the jawbone, and a firm, intimate, and lasting connection between implant and bone can be created (i.e., osseointegration).^1^ A systematic review with meta-analysis showed high 5- and 10- year survival rates of implant-supported fixed prostheses (95.2% and 86.7%, respectively, for fixed dental prostheses; 94.5% and 89.4%, respectively, for single crowns).^2^ However, despite these high survival rates, the same systematic review also reported a frequent occurrence of various types of implant treatment complications (up to 38.7% 5-year complication rate for fixed dental prostheses).^2^

Complications of the implant–suprastructure complex can be biological or technical.^3^ Biological complications affect the peri-implant soft tissues and bone, and are defined by pocket-probing depths, bleeding and/or suppuration on probing, and marginal bone loss over time.^2^ Technical complications affect the mechanical integrity of the implant and suprastructure components, and can be defined into major, such as implant fracture or loss of the prosthesis; intermediate, such as abutment fracture, abutment screw loosening or veneer fractures; or minor, such as loss of retention of the prosthesis, loss of screw hole sealing or chipping of veneering material.^4^

Reporting implant complications is valuable when assessing success of the implant–suprastructure complex as a whole, because, unlike single outcomes such as marginal bone loss or soft tissue parameters, it provides a more comprehensive picture of the total treatment outcome.^5^ Clinical and radiographical evaluations of implants and their suprastructures are generally considered to be important for the detection of early signs of these implant complications.^6^

### Sleep bruxism

Sleep bruxism (SB) has recently been defined as a repetitive jaw-muscle activity characterised by clenching or grinding of the teeth and/or by bracing or thrusting of the mandible during sleep.^7^ It is suggested that jaw-muscle contractions are a natural activity during sleep, and that SB episodes are observed in most individuals.^8^ Up to 37% variability in SB outcome measures has been reported in sleep bruxers,^9^ suggesting that SB has a time-variant nature.

Polysomnography with audio-visual recordings (PSG-AV) is necessary to achieve a definite diagnosis of SB,^7^ according to established cut-off criteria.^10^ However, this technique is costly and often impractical to perform, leading to the use of less accurate methods for diagnosing SB. In clinical practice and research settings, this involves self-report instruments, clinical examinations and electromyographic (EMG) recordings of masticatory muscle activity during sleep.^7,11^

Epidemiological studies based on self-reports have found that ~12.8% of the adult population reports ‘frequent’ SB.^12^ To date, only a single study assessed the prevalence of SB based on PSG recordings and the above-mentioned cut-off criteria in a large population sample.^13^ On the basis of single-night PSG recordings, the prevalence of SB in an adult population was 7.4%, regardless of self-reported bruxism complaints.^13^ In the future, studies implementing diagnostic methods capable of capturing the time-variant nature of SB should lead to more accurate figures about the prevalence of SB.^9^ Prevalence data based on sound criteria are important. However, the clinician interested in the consequences of SB should be aware that, even individuals who, after one or several PSG recordings, would not officially be characterised as sleep bruxers, can present (mild) bruxism activity during sleep.^14^

### SB and implant treatment complications

SB is considered an important source of loading applied to implants and their suprastructures, and it is a longstanding concept that SB can lead to biological and technical complications.^3^ Two recent systematic literature reviews point towards the notion that bruxism can contribute to the occurrence of mainly technical failures of implant treatments,^15,16^ while there is no sound evidence that bruxism is related to biological implant complications.^15^

However, a causal relationship between SB and (peri-)implant complications has not yet been demonstrated.^15,16^ This lack of sound evidence is the consequence of mainly two factors. First, up to the present time, there is no study with the specific design to assess the effect of bruxism on dental implants. Studies that have included bruxism as one of the factors contributing to complications show a large variation in terms of both the technical and the biological outcomes of implant treatments, so that their comparability is compromised.^15,16^ Second, there are issues regarding the internal validity of those studies, such as an inadequate distinction between sleep and awake bruxism, and insufficient diagnostic approaches of SB.^3,15,16^ On the basis of the assumption that SB can lead to complications, clinicians are so far instructed to be cautious, and are guided by expert opinions regarding practical aspects of implant treatments in patients with (suspected) bruxing behaviour.^15^ These guidelines include advices on implant and suprastructure characteristics, such as the number, length and diameter of implants, the material of the suprastructure, and the occlusion and articulation patterns.^3^ Expert opinions represent the lowest grade of evidence and cannot, therefore, fulfil modern clinicians’ needs for evidence-based decision-making.

The lack of high-level evidence also affects researchers, as suspected bruxism is often—but not always—an exclusion criterion in studies concerning the outcomes of dental implant treatments.^3^ Consequently, the populations in such studies may significantly differ from each other, which undermines the ability to compare their outcomes in an unequivocal way. Furthermore, if SB proves indeed to have detrimental consequences for the success of implant treatments, it is an important factor to be considered when designing relevant studies.

### Mechanical loading and biological parameters

Clinical studies, literature reviews and expert consensus papers^17–19^ report that formation and maturation of a microbial biofilm around an implant is an important aetiologic factor in the pathogenesis of the peri-implant infectious diseases ‘mucositis’ (i.e., inflammatory process in the mucosal tissue) and ‘peri-implantitis’ (i.e., inflammatory process additionally characterised by marginal bone loss). Submucosal biofilm associated with these diseases has been reported to present low species diversity, with fewer numbers of bacterial species found around diseased implants, compared to the biofilm found around healthy implants.^20^ Other factors related to the occurrence of peri-implant disease include smoking and a history of periodontitis.^21^

On the basis of current literature, it is not fully known whether and how mechanical implant loading contributes to peri-implant tissue complications (i.e., inflammation and bone loss). Several clinical studies^22,23^ suggest that high mechanical stress, exceeding the biological load-bearing capacity of an osseointegrated oral implant,^24^ is associated with loss of marginal bone or even loss of the osseointegration around the implant. More recently, a review on animal studies found differences in the histological features between plaque- and overload-induced peri-implant bone loss.^25^ However, as yet, causative relationships between mechanical loading and peri-implant biological complications have not been established, due to a general lack of clinical studies with an appropriate design to assess the effect of excessive loading on dental implants,^26^ and poor definitions of the loading conditions (e.g., poor approaches to diagnose parafunctions such as SB).^15^

It remains unclear if, and to what extent, loading and microbial factors interact in the process of peri-implant tissue destruction.^24^ Animal experimental data suggest that high loading of clinically stable dental implants is associated with marginal bone loss in the case of inadequate plaque removal, while when plaque control is sufficient, this loading might lead to an increase of bone density around the implant.^27^ In addition, data revealing unique and unsuspected microbial communities around failing implants have recently been presented,^20^ whereas sparse human clinical data suggest a possible different microbial profile between implants failing due to mechanical overload, as compared to implants failing due to peri-implant infection.^28,29^ However, thus far, research supporting these suggestions is not conclusive.^30^ Investigating the time-dependent associations between mechanical forces (such as those attributed to SB), the composition of microbial communities and host response will enhance our insight into the pathogenesis of peri-implant disease.

### Objective of the present study

To contribute to the understanding of the time-dependent associations between SB and complications of dental implant treatments, we aim to perform a prospective cohort study. Our main aim is to answer the research question: ‘Is sleep bruxism a risk factor for (peri-)implant complications?’. SB will be monitored by measuring masticatory muscle activity during sleep. The investigation will have two main outcomes, namely, technical complications and biological complications. As to avoid variation in the outcomes caused by failing retention of removable suprastructures, we will confine our study population to patients treated with fixed suprastructures.

The following null hypotheses are formulated: (1) Sleep bruxism is not associated with the occurrence of technical complications. Outcomes of interest are: suprastructure complications, abutment complications, implant fractures or other technical failures (see ‘Variables’ paragraph for a more comprehensive description of all variables), and (2) SB is not associated with the occurrence of biological complications. Main outcomes related to this hypothesis are: differences in marginal bone height, peri-implant bleeding on probing, pocket depths and loss of osseointegration.

Our secondary aim is to examine whether there is an association between SB activity and the composition of peri-implant submucosal biofilm. For this purpose, the null hypothesis is: SB is not associated with species diversity of peri-implant submucosal biofilm. The main outcome will be peri-implant submucosal microbiome diversity.

### Study design

The study has a prospective, double-blind design and will be performed in the Academic Centre for Dentistry Amsterdam (ACTA), The Netherlands. Aimed duration of the study is 3 years: 1-year sampling period and 2 years of follow-up. Participants of the study will receive one or more dental implants, which will be loaded with a fixed dental prosthesis for replacement of one or more lost teeth. Baseline assessment (T_1_) of each participant will take place after the healing period of the implant(s), at the appointment of taking the impressions for the prosthesis. Afterwards, assessments will take place within a follow-up period of 2 years at pre-fixed time intervals (2 weeks; T_2_, 6 weeks; T_3_, 3 months; T_4_, 12 months; T_5_, and 24 months after baseline; T_6_, see Table 1). These assessment intervals match those of the regular clinical procedures at ACTA, with the exception of T_3_, which represents an additional examination. Recurrent ambulatory EMG recordings for the diagnosis of SB will be performed by the participants in their home environment at T_1_, T_3_ and T_5_. Clinical measurements will be performed by one examiner (MT) at ACTA. This examiner, as well as the participants, will be blinded for the main predictor of the study, i.e., SB diagnosis based on EMG recordings.

## Materials and methods

### Ethical considerations and registration of study

All study procedures are performed according to the guidelines issued in the Declaration of Helsinki. Approval of the research protocol by the Medical Ethical Committee of the Vrije Universiteit Amsterdam was obtained (METC—VUmc ref.: 2011-245). The Dutch Healthcare Inspectorate acknowledged that the obligation for notification of the Dutch Healthcare Inspectorate prior to the start of the clinical investigation was fulfilled. The research is included in the Netherlands Trial Register (Trialregister.nl, reference no.: 4930) and is registered at the US National Institutes of Health (ClinicalTrials.gov Identifier: NCT02410681). Extensive documentation of all standard operating procedures is performed, following ISO 14155.2011 criteria. Collected data will be digitally stored using ALEA Data Management-version 4 (ALEA Data Management, FormsVision, Abcoude, The Netherlands). ALEA provides online data management tools for use in clinical trials and enables tracking of all changes made to previously inserted data.

### Participants

Participants will be enrolled in the study if they fulfil the inclusion and exclusion criteria, agree to participate, and sign the informed consent form. Participants will be recruited from the clinic of the Department of Oral Implantology and Prosthetic Dentistry of the ACTA. This department treats patients situated in the greater Amsterdam area. Inclusion criteria are: a planned treatment with implant-supported fixed suprastructure(s) and age 18 years or older. Exclusion criteria are: opposing teeth of implant-supported fixed suprastructure(s) are restored with removable artificial teeth; patients categorised in the classes 3 or higher according to the American Society of Anesthesiologists (ASA) system for classification of physical status;^31^ use of an occlusal splint, mandibular repositioning appliance or any other bruxism mitigating device during sleep; active periodontitis at the time of implant placement; known allergy to the EMG device electrode material; usage of a pacemaker; and swollen, infected or inflamed tissues or skin eruptions, e.g., phlebitis, varicose veins etc. in the placement area of the EMG device electrode. Pregnant women will not be treated with dental implants. Pregnancy after the placement of implants will not be a reason to stop participation of the subject in the study.

All patients of the clinic of Oral Implantology and Prosthetic Dentistry for which one or more implants are planned will be screened for fulfilment of the inclusion and exclusion criteria and willingness to participate in a clinical study. Eligible patients will be thoroughly informed about the study, upon which they will be given 1-week time to consider participation. Written informed consent will be obtained prior to enrolment of a patient in the study. Participation is voluntary and can be withdrawn at any time, without consequences for the course of treatment.

### Sample size

To analyse the association between predictors and outcome variables for both our main aim and our secondary aim, multilevel regression analyses will be used.^32^ Therefore, we will use the suggested formula ‘50+8*k* (*k*; number of predictors)’ for the calculation of our sample size.^33^ On the basis of that formula, this study (with six predictors) will need a minimum sample size of ‘50+48=98’ participants.

### Variables

An overview of the time points at which each variable is assessed is provided in Table 1.

#### Main predictor

##### Sleep bruxism

Sleep laboratory PSG-AV together with self-report and clinical examination is currently considered to lead to a definite diagnosis of SB.^7^ PSG-AV recordings enable quantification of SB-specific muscle activity, i.e., rhythmic masticatory muscle activity of masseter and/or temporal jaw muscles, as well as exclusion of non-SB-specific muscle activity, e.g., swallowing and scratching.^34^ However, it is difficult to use the PSG-AV for large sample studies due to feasibility and financial considerations.^35^

As alternatives to PSG-AV, various types of ambulatory recorders of masticatory muscle activity have been developed. Those have the obvious benefits of a natural home setting and low costs, and have been used in clinical studies, although specificity of the SB-specific muscle activity assessment remains a limitation.^35^ Therefore, muscle activity assessed with ambulatory recorders is considered a proxy for a SB diagnosis.

In our study, SB is assessed by measuring the EMG activity of the right temporal muscle during sleep with an ambulatory EMG recorder (Grindcare, version 3+ DL, Delta Danish Electronics, Light & Acoustics, Hørsholm, Denmark), at the home setting of the individual.^35^ Grindcare is a device designed for the management of SB and consists of a sensor (the portable unit which registers EMG activity and can be attached to the individual’s clothing) and the electrode (which attaches to the skin over the temporal muscle and is connected with the recording device by a wire). It can detect and record muscle EMG activity and issue a weak electrical stimulus on the skin, aimed at eliminating bruxism activity. For the purposes of this study, the issue of electrical stimuli is turned off and the device is used in its diagnostic mode. Within the Grindcare 3+ DL device, the EMG signal is amplified 808 times and bandpass filtered between 200 and 650 Hz. The signal is then converted by an analogue-to-digital converter within a range of 0–1.5 V and stored on a microSD card, from which it can be transferred and stored on an personal computer for further analysis. There are three sessions of sleep recordings (T_1_, T_3_ and T_5_), each consisting of three consecutive nights. The first session will start at the day of the baseline measurements. The second session takes place at 6 weeks from baseline, and the third session at 12 months from baseline. During their presence in the clinic, participants will be thoroughly trained on the function of the Grindcare device and placement of the electrode on the area of greatest distension of the right temporalis muscle by one examiner (MT). In addition, they will be provided with a clearly written and illustrated instruction manual to aid in the proper use of the device. At the start of each recording, participants are instructed to perform three maximum voluntary clenches in maximum intercuspation, each lasting for at least 3 s, with 10 s of rest between them. Within 2 weeks after each recording session, the device is returned to the examiner (MT), who will transfer the raw EMG data to a personal computer. The EMG signal will be assessed for the presence of an acceptable signal-to-noise ratio (i.e., >10), during a sufficient length of the recording (i.e., at least 75% of the length of the recording), and absence of artefacts, such as loss of the electrode, by custom-made software, designed for this purpose by the software engineer of the department of Oral Kinesiology of ACTA. If one or more recordings fail, they will be repeated as soon as possible, and no longer that 2 weeks after the first raw EMG data have been evaluated.

The raw EMG data will be analysed for the calculation of SB outcomes upon completion of the entire follow-up period of the study, using a stepwise analysis tool incorporated in the BruxismDetector software. This software has been developed at the Department of Oral Kinesiology of ACTA. The beginning of the sleep period will be defined as 30 min after electrode placement, and the end at moment when the EMG signal starts to exhibit an unstable pattern prior to electrode removal. During total sleep time, EMG amplitudes >20% of the highest maximum voluntary clenche will be selected for SB episode scoring, according to the Lavigne *et al.* ^10^ criteria. Type of SB episodes will be scored as follows: phasic (at least three suprathreshold EMG bursts lasting ⩾0.25 and <2.00 s, and separated by two interburst intervals of <2.00 s), tonic (one EMG burst lasting⩾2.00 s) or mixed (both phasic and tonic types of bursts). When the time interval between two bursts is ⩾2.00 s, a new episode is considered to start.^10^ Per recording, two SB outcome variables will be derived, viz., the number of bruxism episodes per hour of sleep (Epi h^−1^), and the bruxism time index (i.e., the total time spent bruxing divided by the total sleep time, times 100%).^9,36^

#### Outcomes

##### Biological complications

Biological complications will be assessed by examining cardinal features of peri-implant health, i.e., bleeding on probing, probing depths and marginal bone height.^37^ Also, loss of osseointegration will be registered.

Peri-implant bleeding on probing will be scored per implant according to the Modified Gingival Index^38^ as follows:

- Score 0; no bleeding when a periodontal probe is passed along the gingival margin adjacent the implant;
- Score 1; isolated bleeding spots visible;
- Score 2; blood forms a confluent red line on margin;
- Score 3; heavy or profuse bleeding.

Peri-implant probing depths will be scored using a standardised periodontal probe (Click-Probe 3/5/7/10 blue, KerrHave, Bioggio, Switzerland) with pressure of 0.2–0.25 N. Clinical probing depth will be measured in millimetre as the distance from the mucosal margin to the bottom of the deepest clinical probing site on each side of the examined implant (mesial, distal, buccal and lingual). Per implant, the mean value of those sides is calculated.

Marginal bone height will be assessed radiographically. Vertical bitewing radiographs will be taken using the parallel cone technique and phosphor plates (VistaScan Image Plate, Dürr Dental, Bietigheim-Bissingen, Germany), with the assistance of individually modified plate positioning devices. Modification of the positioning devices will aim at acquiring geometric reproducibility of the radiographs during the successive examinations of the participants. More specifically, reproducing the intraoral position of the device will be achieved by using a silicone (Provil Novo, Putty regular set, Heraeus Kulzer, Hanau, Germany) mould, made just after the suprastructure is placed. A reproducible position of the X-ray tube with respect to the plate positioning device will be acquired by the use of a customised hard plastic aiming block. Radiographs are taken with a dental X-ray generator operated at 63 kV DC, 8 mA, and exposure time of 0.32 s. The obtained images will be imported in a commercial dental image archiving program (Emago, Oral Diagnostic Systems, Amsterdam, The Netherlands). Marginal bone height will be measured on each radiograph using the appropriate tool of the program as the vertical distance (mm) from a fixed landmark point on an implant (implant shoulder) to the superior border of the marginal bone at each of the mesial and distal sides of the implant. The Subtraction technique of the Emago software will be used to detect differences in marginal bone between subsequent radiographs. Measurements will be performed by two independent examiners. Per implant, the mean value of both sides is calculated.

Mobility of the implants or their suprastructures will be assessed manually by clinical investigation using the back part of the handles of two hand instruments (e.g., mouth mirror and probe). Mobility will be scored as either present or not. When mobility is present, the examiner will investigate and note the cause of mobility, i.e., technical complication(s) or loss of osseointegration.

Throughout the course of the study, if any of the aforementioned conditions require treatment, usual care will be provided.

##### Technical complications

Implant technical complications will be assessed by clinical and radiographical examination. The following complications will be registered:

1. Suprastructure complications (complete or incomplete fracture of veneer, fracture of framework, loosening of occlusal screw or fracture of luting cement, fracture of occlusal screw).
2. Abutment complications (loosening or fracture of abutment screw, fracture of abutment).
3. Implant fracture.
4. Other complications (e.g., loss of occlusal screw seal).

In case any of these complications occur, the treating dentist will be informed and appropriate treatment will take place.

##### Composition of peri-implant submucosal biofilm

The Shannon diversity index will be used for expressing microbiome diversity.^20^ Composition of peri-implant submucosal biofilm will be analysed by means of genome analysis of bacterial samples, using an open-ended sequencing technique.^39^ Per implant, biofilm will be collected after supramucosal plaque has been removed by means of polishing paste (Proxyt fine paste, Ivoclar Vivadent) and a polishing cup, and the clinical crown has been rinsed with water and dried with air. Sterile paper points (Henry Schein Absorbent Points #504 medium, Henry Schein, Melville, NY, USA) and sterile dental tweezers will be used to collect intrasulcular peri-implant biofilm from four sites of each implant (mesial, distal, lingual and buccal). Samples will be transferred to tubes (Axygen Self-standing, clear, sterile Scientific Screw Cap Tubes, 2.0 ml, Axygen, Union City, CA, USA) and stored in the laboratory of ACTA at −80 °C until further analysis.

#### Covariates and confounders

The association between SB and the outcome variables described will be controlled for possible interacting and/or confounding effects from a series of other variables. These include smoking status, self-reported awake bruxism, peri-implant plaque accumulation and periodontal parameters. These variables have been chosen based on literature supporting either their purported modifying influence on the main outcomes (i.e., covariates) or their association with both our main predictor and main outcomes (i.e., confounders), and are described below.

Smoking has been shown to be associated with SB,^40^ peri-implant and periodontal inflammation, and bone loss.^21^ Also, it has been shown to affect both the composition of subgingival^41^ and submucosal^42^ biofilm. Smoking will be evaluated by four categories (never, occasional, former and current), using the questionnaire developed by Hukkinen *et al.* ^43^ Participants reporting smoking <5–10 packs are categorised as never-smokers. Participants having smoked >5–10 packs, but never on a regular basis, that is, daily or almost daily, are categorised as occasional smokers. Regular smoking in the past defines a participant as former smoker, whereas regular present smoking represents current smoker. Other forms of tobacco use (cigars, cigarillos or pipe tobacco) are dichotomised and defined as someone never having smoked any of these forms of tobacco (classified as never-smoker), or having ever smoked at least 50 cigars, 75 cigarillos and/or >3–5 packages of pipe tobacco (classified as current smoker).

Clenching and/or grinding of the teeth while awake are manifestations of awake bruxism^7^ and may form an important source of mechanical loading of implants and their suprastructures. Awake bruxism will be evaluated by a self-report 5-point scale (0=never, 1=rarely, 2=sometimes, 3=often and 4=always), using a single item derived from the Dutch translation of the Oral Behaviors Checklist.^44^ Subjects will be asked: ‘Could you indicate how often you have performed the following activities during the last month: clench or grind your teeth while you are awake?’.

Peri-implant supramucosal biofilm has reported to be crucial for the development of peri-implant disease.^6^ Per implant, plaque accumulation is scored according to the modified Plaque Index^39^ as follows:

- Score 0: no detection of plaque;
- Score 1: plaque only recognised by running a periodontal probe across the smooth marginal surface of the implant. Implants covered by titanium spray in this area always score 1;
- Score 2: plaque can be seen by the naked eye;
- Score 3: abundance of soft matter.

Periodontal parameters of all present natural elements and implants, with the exception of the implants being studied, will be recorded, as periodontal disease has been reported to favour the occurrence of pathology around dental implants.^45^ These parameters include the number of clinical pockets with probing depths of ⩾5 mm and Bleeding Index. Probing depths are measured as the distance from the gingival (or mucosal) margin to the bottom of the deepest clinical pocket on six sides of the examined tooth or implant (mesio-buccal, mesio-lingual, disto-buccal, disto-lingual, buccal and lingual), using a standardised periodontal probe (Click-Probe 3/5/7/10 blue, KerrHave) with pressure of 0.2–0.25 N. Pockets with depths of ⩾5 mm are scored as either present or absent. Bleeding on probing is assessed on the same sides described above, using a standardised periodontal probe (Click-Probe 3/5/7/10 blue, KerrHave) with gentle pressure of 0.2–0.25 N as either present or absent within 30 s after probing. The Bleeding Index (%) is calculated using the formula: (number of sites with bleeding/total number of sites examined)×100.

Morphological and restorative aspects of the implant treatments are registered in the dental record of each subject as part of regular clinical practices of ACTA, and will serve for the description of the baseline characteristics of our subjects’ implants. These include the number of occluding pairs of natural teeth, geometrical characteristics of the implants, factors related to loading of the implants, prosthetic characteristics of the implant-supported suprastructures and the status of their antagonists.

The number of occluding pairs of natural teeth is defined as the number of pairs between upper and lower equivalent natural teeth, by oral assessment. Implant characteristics recorded are the type of implant (manufacturer and system) and implant size (diameter and length, in mm). Characteristics related to loading of the implants and loadability of the receiving bone site include time of loading the implant with the definitive prosthesis (immediate, i.e., within 1 week after implant placement; early, i.e., between 1 week and 2 months; or late, i.e., after 2 months), bone or soft tissue augmentation procedures, bone quality (according to the criteria proposed by Lekholm and Zarb^46^), and position of implant (within arch, lower or upper jaw). Prosthetic characteristics of the implant-supported suprastructures are noted. Type of abutment (material, fabrication method), type of implant-supported suprastructure (single crown, fixed partial denture with or without cantilever), retention type (cemented or screw-retained) and material of the suprastructure are registered. Regarding the antagonists of the studied implants, the structure of opposing occlusal contact(s) (natural tooth, implant or none), type of restorative material present on opposing supporting cusp(s), and occlusal contact of implant-supported restoration with antagonists during maximum intercuspation protrusion and/or laterotrusions are registered after oral assessment, with the aid of a 12 μm occlusal foil (Hanel occlusion foil 12 μm, Coltene, Langenau, Germany).

### Reporting of data and statistical analysis

#### Descriptive statistics

Descriptive statistics for categorical variables will be presented using frequency tables, and continuous variables will be presented as each mean and s.d.^47^ The incidence of biological and technical complications will be reported.^47^

#### Single/multiple analysis

Our hypotheses will be tested using multilevel regression analyses.^32^ As for the main aim of the study, hypotheses about associations between SB and the occurrence of technical complications or loss of osseointegration will be tested by using a Cox proportional hazards regression model.^48^ Possible associations between SB and bleeding on probing, pocket depths and marginal bone height will be tested by means of linear regression analysis. Likewise, for our secondary aim, possible associations between SB and microbiome diversity will be tested by means of linear regression analysis. The regression models will be expanded to examine the effects of confounders and covariates on the associations between main predictor and main outcomes. We aim to adjust our analysis for dependency of data, which arises from the fact that multiple implants can be placed in one subject, thus violating the assumption of independent observations.^49^

## Discussion

To our knowledge, this is the first clinical prospective cohort study to investigate the associations between SB and dental implant treatment complications.

Certain strong points of this investigation should be recognised. First, the study aims to answer a clinically relevant question, for which the literature so far is inconclusive. A strength of our design is the clear distinction between sleep and awake bruxism that is made. Given the possibility that the different types of bruxism may be differently associated with implant failure, together with the fact that these two conditions require different management approaches,^49^ employs that this distinction will enhance the clinical applicability of our results. SB will be diagnosed by means of EMG recordings. Though less valid than the gold standard, i.e., PSG recordings with audio-visual recordings, this feasible diagnostic method is a good proxy, and by performing repeated measurements it enables capturing the time-variant nature of SB. What is more, by extensively documenting our study procedures and the careful data management, we aim to contribute to the performance of transparent and reproducible research.

On the other hand, certain limitations should be acknowledged. For one, no patient-related outcomes, such as satisfaction with the prosthesis, aesthetics or function, will be assessed. Also, no outcomes related to complications-associated costs of treatment will be studied. Though it would be of interest to investigate these parameters, they were not included in our protocol for the sake of avoiding a too complex study design. Future investigations should address these outcomes. Also, our patient sample is derived from a dental school, thus it could be hypothesised that it differs from the population attending other dental care facilities. However, no data are available today to describe if and how these populations might differ from each other.

## Disclaimer

Grindcare is a trademark of Sunstar Suisse SA, which provided the EMG devices for the duration of the study. Sunstar Suisse SA is not involved in the design of this study, nor in the collection, analysis and interpretation of data, and writing of this manuscript.

## Figures and Tables

**Table 1:** Overview of examination time points and variables collected

*Time point (time from baseline)*	*Variables collected*
T_0_ (0)	Sleep bruxism, modified gingival index, peri-implant probing depth, marginal bone height, loss of osseointegration, modified plaque index, submucosal biofilm samples, awake bruxism, smoking status, periodontal parameters.
T_1_ (2 weeks)	Modified gingival index, peri-implant probing depth, marginal bone height, loss of osseointegration, modified plaque index, periodontal parameters.
T_3_ (6 weeks)	Sleep bruxism, modified gingival index, peri-implant probing depth, marginal bone height, loss of osseointegration, technical complications.
T_4_ (3 months)	Modified gingival index, peri-implant probing depth, marginal bone height, loss of osseointegration, technical complications, submucosal biofilm samples.
T_5_ (12 months)	Sleep bruxism, modified gingival index, peri-implant probing depth, marginal bone height, loss of osseointegration, technical complications, modified plaque index, submucosal biofilm samples, awake bruxism, smoking status, periodontal parameters.
T_6_ (24 months)	Modified gingival index, peri-implant probing depth, marginal bone height, loss of osseointegration, technical complications.
